# Decision-Making Process of Home and Social Care Professionals Using Telemonitoring of Activities of Daily Living for Risk Assessment: Embedded Mixed Methods Multiple-Case Study

**DOI:** 10.2196/64713

**Published:** 2025-04-25

**Authors:** Renée-Pier Filiou, Mélanie Couture, Maxime Lussier, Aline Aboujaoudé, Guy Paré, Sylvain Giroux, Hubert Kenfack Ngankam, Patricia Belchior, Carolina Bottari, Kevin Bouchard, Sébastien Gaboury, Charles Gouin-Vallerand, Faustin Armel Etindele Sosso, Nathalie Bier

**Affiliations:** 1 Centre de recherche sur le vieillissement (CdRV) Université de Sherbrooke Sherbrooke, QC Canada; 2 CIUSSS Centre-sud-de-l’île-de-Montréal Centre de recherche de l’Institut universitaire de gériatrie de Montréal Montréal, QC Canada; 3 École de réadaptation Faculté de médecine Université de Montréal Montréal, QC Canada; 4 HEC Montréal Montréal, QC Canada; 5 Faculty of Sciences and Faculty of Medicine and Health Sciences Université de Sherbrooke Sherbrooke, QC Canada; 6 School of Physical and Occupational Therapy Faculty of Medicine McGill University Montréal, QC Canada; 7 Department of Mathematics and Computer Science Université du Québec à Chicoutimi Chicoutimi, QC Canada

**Keywords:** activities of daily living, older adults, cognitive deficits, telemonitoring, clinical decisions, public health care system, home care services, mixed methods, multiple-case study

## Abstract

**Background:**

Older adults with cognitive deficits face difficulties in recalling daily challenges and lack self-awareness, impeding home care clinicians from obtaining reliable information on functional decline and home care needs and possibly resulting in suboptimal service delivery. Activity of daily living (ADL) telemonitoring has emerged as a tool to optimize evaluation of ADL home care needs. Using ambient sensors, ADL telemonitoring gathers information about ADL behaviors such as preparing meals and sleeping. However, there is a significant gap in understanding on how ADL telemonitoring data can be integrated into clinical reasoning to better target home care services.

**Objective:**

This paper aims to describe (1) how ADL telemonitoring data are used by clinicians to maintain care recipients with cognitive deficits at home and (2) the impact of ADL telemonitoring on home care service delivery.

**Methods:**

We used an embedded mixed methods multiple-case study design to examine 3 health institutions located in the greater Montreal region in Quebec that offer public home care services. An ADL telemonitoring system—Innovative Easy Assistance System–Support for Older Adults’ Autonomy (Soutien à l’autonomie des personnes âgées in French)—was deployed within these 3 health institutions for 4 years. Subcases (care recipient, informal caregiver, and clinicians) were embedded within each case. For this paper, we used the data collected during interviews (45-60 min) with clinicians only. Quantitative metadata were also collected on each service provided to care recipients before and after the implementation of NEARS-SAPA to triangulate the qualitative data.

**Results:**

We analyzed 27 subcases comprising 29 clinicians who completed 57 postimplementation interviews concerning 147 telemonitoring reports. Data analysis showed a 4-step decision-making process used by clinicians: (1) extraction of relevant telemonitoring data, (2) comparison of telemonitoring data with other sources of information, (3) risk assessment of the care recipient’s ADL performance and ability to remain at home, and (4) maintenance or modification of the intervention plan. Quantitative data reporting the number of services received allowed the triangulation of qualitative data pertaining to step 4. Overall, the results suggest a stabilization in monthly services after the introduction of the ADL telemonitoring system, particularly in cases where the number of services were increasing before its implementation. This is consistent with qualitative data indicating that, in light of the telemonitoring data, most clinicians decided to maintain the current intervention plan rather than increase or reduce services.

**Conclusions:**

Results suggest that ADL telemonitoring contributed to service optimization on a case-by-case basis. ADL telemonitoring may have an important role in reassuring clinicians about their risk management and the appropriateness of service delivery, especially when questions remain regarding the relevance of services. Future studies may further explore the benefits of ADL telemonitoring for public health care systems with larger-scale implementation studies.

**International Registered Report Identifier (IRRID):**

RR2-10.2196/52284

## Introduction

### Background

Cognitive impairments in older adults are closely tied to difficulties in performing activities of daily living (ADLs), resulting in a decline in functional independence [1,2]. This decline increases the risk of neglecting hygiene, forgetting medication intake, and engaging in behaviors that may lead to adverse events such as falls and kitchen fires [3]. The functional decline also contributes to a growing demand for home care support services, which may not be optimized due to the inherent challenges in evaluating the needs of this population [4]. Older adults with cognitive deficits face difficulties in recalling daily challenges and lack self-awareness, amplifying the challenges involved in obtaining reliable information on the areas of functional decline [5]. Home care intervention plans for this population, especially for those living alone, are thus often based on managing *potential* risks, leading to services being implemented to meet anticipated needs, even if not well captured or documented [3,5]. However, the result may be suboptimal service delivery [6], with some older adults receiving too many services and others receiving too few. In addition, many Western countries, including Canada, face a chronic shortage of human and financial resources for public home care services, limiting access to these services [4]. In the context of a rapidly aging population, the optimization of home care services has therefore become a high priority in Canada.

One approach to optimizing services may be to better evaluate home care needs such that the right service can be delivered to the right person at the right time. The telemonitoring of ADLs has emerged as a tool for this optimization. Using diverse sensors such as wearables or ambient sensors (eg, motion detectors, contact sensors, and radio frequency identification), it becomes possible to gather information about an individual’s ADL behaviors within and outside of their home [7]*.* This comprehensive information provides patterns of the ADL routine, including activities such as entering the kitchen, opening and closing cupboards and drawers, and using small electrical appliances. Activity recognition [8,9] is used to derive these ADL patterns, allowing insights into daily, weekly, or even yearly routines [10]. The detection of unusual activity, performance errors, or the absence of movement [11] can indicate inadequately performed activities, highlighting the need for home care support.

Promising results regarding ADL telemonitoring for older adults’ home support have been published in the last 20 years. To provide an update on the current evidence, in 2021-2022, our team conducted an umbrella review [7,12] of 17 published systematic reviews. The review highlighted that ADL telemonitoring has been shown to have moderate evidence of effectiveness in using ADL patterns as an indicator of independent living (eg, as the capacity to perform ADLs). However, because most published systems to date have not yet attained high levels of technology maturity, there is a scarcity of real-life implementation studies and therefore a lack of documented potential benefits for older adults, their caregivers, and health care professionals [7]. There is also a significant gap in the comprehension of how ADL telemonitoring data are integrated into clinical reasoning to better target home care services’ needs and modify or adapt service delivery.

To address this gap, our team conducted a project in close collaboration with the health care system in Quebec between 2016 and 2022. The project was named the Support for Older Adults’ Autonomy program (Soutien à l’autonomie des personnes âgées in French; SAPA), and its overall objective was to codevelop and implement an ADL telemonitoring system to offer better targeted public home care services to older adults living with cognitive deficits in the community. The ADL telemonitoring system developed through this transdisciplinary project was named Innovative Easy Assistance System (NEARS)–SAPA and consists of a lightweight edge computing platform for real-time monitoring installed in the older adults’ homes [13].

The SAPA project involved the close collaboration of all stakeholders in public home care services in the province of Quebec, that is, health institution administrators, heads of services, and health and social care professionals (HSCPs), as well as older adults and their informal caregivers [10]. More specifically, to ensure the development of a relevant, usable, and sustainable technology, we conducted an action design research (ADR) project, comprising a four-stage highly collaborative process [14]: (1) problem formulation; (2) building, intervention, and evaluation; (3) reflection and learning; and (4) the formalization of learning. ADR is generally composed of multiple development cycles, which involve repeating stages 2 and 3 in a cyclical manner. Conducted collaboratively with all stakeholders, these cycles facilitate the refinement of technology and associated interventions for subsequent implementation in real-world settings.

The SAPA project underwent 2 iterative technological design cycles. The first cycle occurred from early 2016 to mid-2018, while the second cycle spanned from late 2018 to late 2022. In the first cycle, we tested a prototype of NEARS-SAPA with a small pool of participants at a health institution located in the greater Montreal region in Quebec, Canada aiming to assess its feasibility and clinical viability [10,15-17]. In the second cycle, we aimed to further improve NEARS-SAPA, as well as its deployment, evaluation, and implementation, in partnership with 3 health institutions also located in the greater Montreal region. This study reports on the data collected during the second cycle of technology development.

### Objectives of This Study and Hypothesis

This paper aims to describe (1) how the ADL telemonitoring data were used by HSCPs in the process of maintaining care recipients with cognitive deficits at home and (2) the impact of ADL telemonitoring on service delivery. We hypothesized that ADL telemonitoring would contribute to service optimization, in that it would enable HSCPs to better identify the types of services their care recipient would need throughout the evolution of their condition (eg, adding an intervention to their clinical plan).

## Methods

### Design

**Figure 1:**
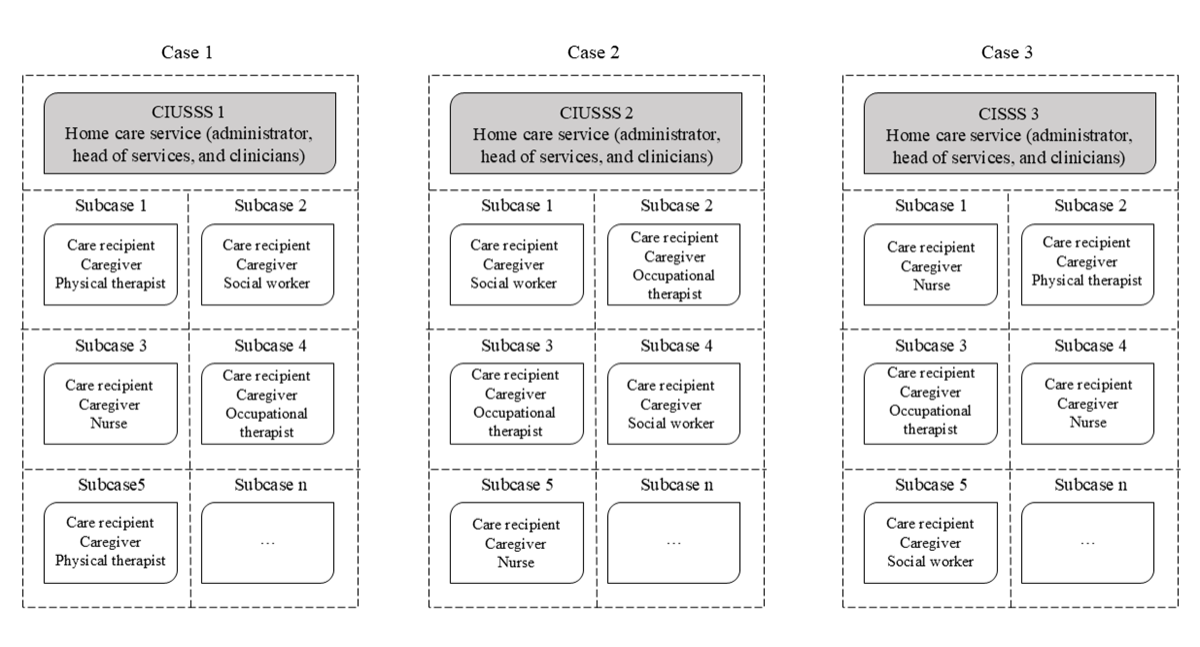
Examples of cases and their embedded subcases. CISSS: centre intégré de santé et de services sociaux (integrated health and social services center); CIUSSS: centre intégré universitaire de santé et de services sociaux (integrated university health and social services center).

To answer our objectives, we used a case study design [18]. A case study is an empirical method that investigates a contemporary phenomenon (the “case”) in depth and within its real-world context [18]. This research design aims to generate theoretical propositions applicable to similar contexts. More specifically, we used an *embedded mixed methods multiple-case study design* [18] in which the cases of interest were 3 health institutions located in the greater Montreal region. Multiple-case studies follow a replication design [18]. In this study, each of the 3 cases was selected so that the individual case studies predict similar results (ie, a literal replication). Within each health institution case, we *embedded subcases*, which included a care recipient, an informal caregiver, and all HSCPs in charge of the care recipients’ care throughout the duration of the study (Figure 1).

In-depth understanding of real-world cases entails the involvement of multiple sources of evidence, with data needing to converge in a triangulating fashion [18]. To answer our objectives, we collected both qualitative and quantitative data using an embedded mixed methods design to combine data [19]. To answer objective 1 (ie, describe how the ADL telemonitoring data were used by HSCPs in the process of maintaining care recipients with cognitive deficits at home), we used the qualitative data collected during interviews with the HSCPs. Although subcases included care recipients and informal caregivers, the data pertaining to HSCPs were most relevant to deepen our understanding of how the data generated by the ADL telemonitoring system were used in practice and how the use of these data impacted HSCPS’ decision-making regarding risk assessment. To answer objective 2 (ie, describe the impact of ADL telemonitoring on service delivery), we used two sources of data: (1) qualitative data collected during interviews with HSCPs and (2) quantitative metadata on services provided to care recipients by the 3 health institutions. We then triangulated quantitative data with qualitative data [20] to assess how the telemonitoring reports had influenced the number of services delivered before and after the introduction of NEARS-SAPA.

This paper used the COREQ (Consolidated Criteria for Reporting Qualitative Research) checklist to promote complete and transparent reporting and improve the rigor and comprehensiveness of qualitative studies.

### Ethical Considerations

The project was approved by the Aging-Neuroimaging Ethical Review Board of the *centre intégré universitaire de santé et de services sociaux* (CIUSSS; integrated university health and social services center) South Central Montreal (CER NV 17-18-10).

HSCPs who met the study inclusion criteria (refer to the Recruitment Procedures subsection) initiated contact with the care recipient to obtain verbal consent for a home visit with a member of the research team. During this visit, the research team member obtained informed and written consent from both HSCPs and their care recipients. When there were changes in HSCPs, the newly appointed professional was made aware of the care recipient’s participation in the study, what their participation entailed for the care recipient and themselves, and asked whether they wanted to pursue this participation. If they agreed to participate, they signed the consent form.

To protect the identity of the care recipients and maintain their confidentiality, they have been assigned fictitious names throughout this paper.

### Descriptions of Cases

#### Overview

In Quebec, the public health and social services institutions are integrated into large health and social services centers named *centre intégré de santé et de services sociaux* (CISSS; integrated health and social services center); or, if affiliated with a university, CIUSSS [21]. Along with *centres locaux de services communautaires* (CLSCs; local community service centers), these health establishments are responsible for delivering care and services to the population of an assigned territory [22]. CLSCs offer frontline health and social services, including home care, through dedicated programs such as the SAPA [21]. The CISSS and CIUSSS offer four types of care services: (1) professional services (eg, nursing, occupational therapy, and physiotherapy); (2) home care services, including assistance with basic and instrumental ADLs; (3) services to informal caregivers; and (4) technical support (eg, medical and specialized supplies and assistive technologies) [23,24]. When an older adult is eligible to receive services, a detailed needs assessment is conducted by a professional to determine the intervention plan, that is, the organization of an individualized service in response to needs [23]. Services are then delivered by various categories of employees depending on the needs. Needs are reassessed over time to adjust the intervention plan in response to new needs or changing conditions. The ADL telemonitoring system was developed to support the home care needs assessment in particular.

#### Case 1: CIUSSS 1

CIUSSS 1 is located on the island of Montreal. Some 15,000 professionals work within CIUSSS 1’s health and social services establishments and serve a population of >300,000 people. The older adult population (aged ≥65 y) accounts for 12.9% of its care recipients, with 47.7% living alone, the highest proportion in Montreal. Of these, 39% also live below the low-income cutoff. The overall population of CIUSSS 1 carries a heavy burden of chronic diseases and reports higher hospitalization rates than elsewhere in Montreal. Half of the overall population (50.7%) has a university degree. The mother tongue of less than a quarter (22.1%) of the population is not an official language of Canada (French or English), and 1.7% speak neither official language.

#### Case 2: CIUSSS 2

CIUSSS 2 is also located on the island of Montreal. Some 14,000 professionals work within CIUSSS 2’s health and social services establishments and serve a population of >375,000 people. The older adult population (aged ≥65 y) accounts for 19% of its care recipients, with 27.8% living alone. Of these, 22.5% live below the low-income cutoff. More than a third (37.3%) of the overall population has a university degree. The mother tongue of close to a third (32.4%) of the population is not an official language of Canada (French or English), and 1.9% speak neither official language.

#### Case 3: CISSS 3

CISSS 3 is located in the greater Montreal region. Some 13,000 professionals work within CISSS 3’s health and social services establishments and serve a population of >405,000 people. The older adult population (aged ≥65 y) accounts for 18% of its care recipients, with nearly one-third living alone, while 1 in 2 older adults lives with at least 1 chronic disease. Materially and socially advantaged as a whole, only 6.9% of CISSS 3’s overall population live below the low-income cutoff, and only 16% of people (aged ≥25 y) have no university certificate, diploma, or degree. Anglophones represent 14% of the population.

### Description of the NEARS-SAPA Telemonitoring System

**Figure 2:**
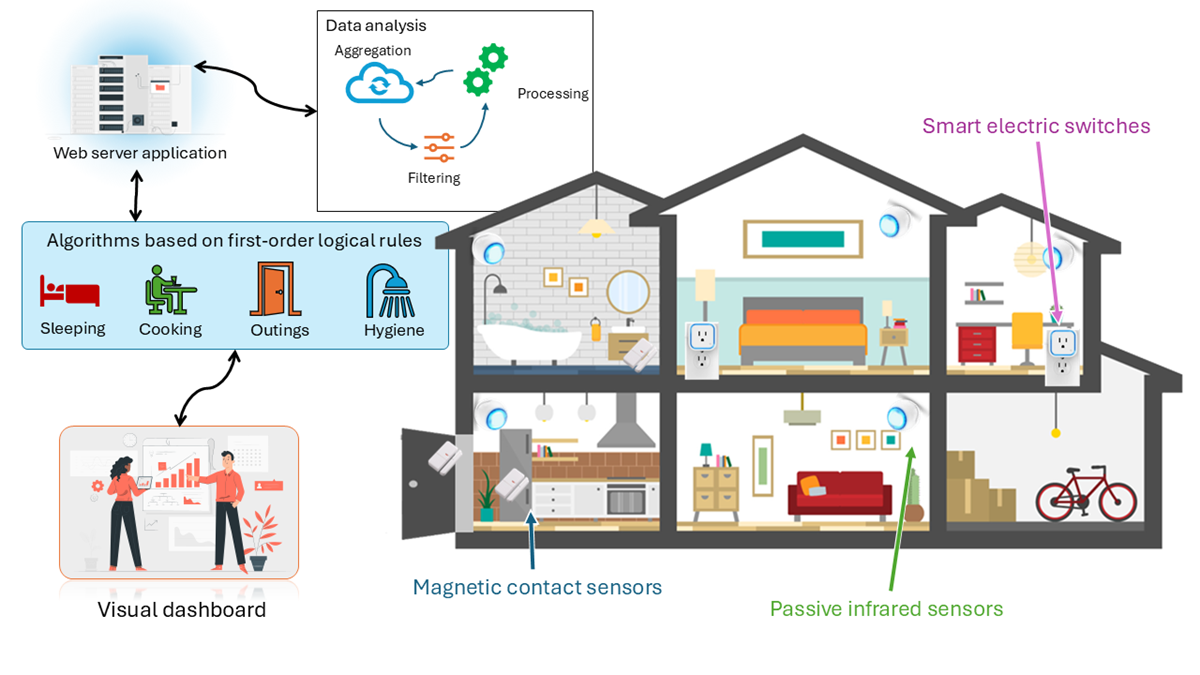
The Innovative Easy Assistance System–Support for Older Adults’ Autonomy program (Soutien à l’autonomie des personnes âgées in French; NEARS-SAPA) telemonitoring system.

The NEARS-SAPA ADL telemonitoring system (Figure 2) comprises a set of ambient nonintrusive sensors, installed in different rooms of the care recipient’s home, thus allowing a portrait to be drawn of the activities performed by the individual [13,15,25,26].

Three types of sensors were used: (1) passive infrared sensors, (2) magnetic contact sensors, and (3) smart electric switches. In general, for a 3-room house, we installed 7 to 8 motion detectors, 9 to 10 contact sensors, and 2 to 3 electrical sensors. Due to the nature of these sensors, which monitor overall activity levels, NEARS-SAPA is best suited for individuals living alone. Algorithms based on first-order logical rules were developed to track five key activities: (1) sleeping, (2) outings, (3) cooking, (4) personal hygiene, and (5) general activity level in the home. Each activity recognition relies on an aggregation of data from ≥2 sensors to produce high-level knowledge of activities, such as the presence of a person in a room or the use of a specific device. The system also captures the performance of an action, such as opening a door. From these algorithms, graphical representations and statistics for the care recipient’s daily routines are generated in the form of a visual dashboard, all accessible through a secure web application. The system thus performs data acquisition and analysis but does not make recommendations in terms of decisions and actions and does not send notifications [10].

**Figure 3:**
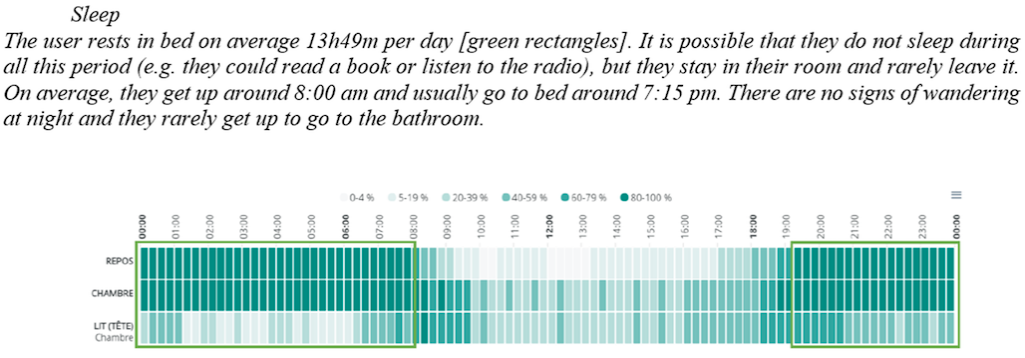
Example of a telemonitoring report of activities of daily living sent to health and social care professionals taking part in the Support for Older Adults’ Autonomy program (Soutien à l’autonomie des personnes âgées in French; SAPA).

In response to the COVID-19 pandemic, modifications were made to NEARS-SAPA and to the project methodology. This served to comply with health and safety regulations as well as accommodate the limited availability of HSCPs amid heightened demand. Hence, due to restricted in-person access to health care institutions, the installation of secure pathways and certifications on HSCPs’ computers was unfeasible. Instead of relying on the web application as initially intended, the research team compiled data from various sensors into PDF reports and sent them directly to HSCPs via email (Figure 3). Each report included a user guide on algorithm calculation and data description, followed by graphical representations and accompanying descriptions of activity trends. Approximately 1 month after sensor installation, HSCPs received the first report detailing the care recipient’s activity patterns during the preceding month. Subsequent reports were issued approximately every 2 months, allowing professionals to track trends and changes in ADLs among the care recipients under their care, without notifications being sent as requested by the HSCPs [10].

### Recruitment Procedures

Purposive sampling was used to achieve an in-depth understanding of the phenomenon under study [27]. When using purposive sampling in qualitative research, reaching data saturation requires a strategic approach to selecting participants and continually analyzing the data (refer to the Qualitative Analysis subsection).

Researchers intentionally select participants or cases that are most relevant to the research question or that can provide the most insightful, rich, or deep data based on specific characteristics, experiences, or expertise. The goal is not to achieve statistical representativeness but rather to ensure that the sample includes individuals who can offer meaningful and relevant information to address the research objectives. Within each of the 3 sites, administrators or heads of services were tasked with requesting their clinical teams to identify the care recipients who might benefit from ADL telemonitoring and refer them to the research team. After receiving a presentation of the project at their workplace, interested HSCPs filled out a request form and discussed their ADL telemonitoring needs with the research coordinator (ML) by email. To be recruited into the SAPA project, HSCPs had to refer care recipients (1) who had experienced a loss of autonomy requiring home care services, (2) who lived alone, and (3) for whom their clinical concerns and questions fell within the technological constraints of NEARS-SAPA. In this study, we included HSCPs who completed at least 1 interview after the implementation of NEARS-SAPA at the care recipient’s home.

### Data Collection

#### Qualitative Data: Semistructured Interviews With HSCPs

Interviews with HSCPs were conducted over the telephone or via videoconference by a member of the research team (AA) trained in qualitative research. Interviews took place during HSCPs’ working hours and lasted 45 to 90 minutes. The following questions were asked:

1. “Overall, how did you use the telemonitoring data?”
2. “Can you identify the activities of daily living for which you used the telemonitoring information?”
3. “Please describe the type of information that was useful to you.”
4. “How did the telemonitoring data influence the intervention plan for the care recipient?”

The first postimplementation interview was conducted after the HSCP had received 2 ADL reports, then after every 2 reports sent (when possible) or until the end of the care recipient’s participation (ie, the end of the project, relocation, or death). Therefore, the HSCPs were included in the study as long as their care recipient had the system installed in their home.

Changes in HSCPs occurred frequently throughout a care recipient’s participation in the study. When this happened and if the newly appointed professional agreed to pursue participation and provided consent, the research team sent them all available ADL telemonitoring reports, and the interviewer then proceeded to interview the new clinician. It is worth adding that a few HSCPs had more than 1 care recipient participating in the study. In these rare cases, the interviewer would discuss all older adults under the HSCP’s care, sequentially, during the same interview. However, ADL telemonitoring reports remained individualized.

All interviews were audio recorded and transcribed verbatim by a person specialized in this type of work. They were further validated by a member of the research team. All verbatim extracts included in this paper were translated from French into English by a bilingual member of the research team (RPF).

#### Quantitative Data: Clinical Services Delivered to Care Recipients

We collected metadata on each service provided to care recipients by the CIUSSS and CISSS as recorded in the ICLSC, which is the Quebec ministerial database that contains information and stores data on service users, requests, and interventions provided by the institutions. The database is used to describe frontline services to ensure the quality and efficiency of health and social services. For this study, we examined the monthly count of services related to ADLs received by the care recipients over a span of 12 months: 6 months before and 6 months after receiving the first NEARS-SAPA telemonitoring report. The services provided were aggregated on a monthly basis and numbered with reference to the introduction of ADL telemonitoring reports for HSCP. In this way, months 1 to 6 represented the initial period, while months 7 to 12 denoted the period after the introduction of telemonitoring reports. Each care recipient was treated as a longitudinal subcase.

For all participants, we collected the latest Iso-Système de mesure de l’autonomie fonctionnelle (Iso-SMAF) profiles available in the care recipient medical database. The Iso-SMAF profiles [28] are a decision-making tool designed to guide individuals toward the resources appropriate to their needs. Their ultimate goal is to support clinical judgment, and they are widely used in all health and social service establishments across Quebec. When available (not systematically administered), we also collected the Mini-Mental State Examination (MMSE) [29] and Montreal Cognitive Assessment (MoCA) [30] scores, which are used to assess cognitive function, particularly in the context of diagnosing cognitive impairments.

### Data Analysis

#### Qualitative Analysis

Qualitative analysis allows for a rich understanding of a focused and bounded phenomenon in a specific context [31]. In this study, the phenomenon is the use of ADL telemonitoring information in the clinical decision-making process of HSCPs in the context of home care services. Data analysis was performed using the method described by Miles [31]. More precisely, memos, coding, and matrix building were used. When analyzing a postimplementation interview with an HSCP, a memo was written to describe their decision-making process in detail for each activity discussed with the interviewer. Data analysis was performed following a deductive-inductive approach, using the framework of integration of ambient assisted living monitoring technologies within clinical decision-making developed by Lussier et al [15]. The framework was supplemented by other relevant components that emerged from our data. Data analysis was first performed separately for each CISSS and CIUSSS to identify similarities and differences. As no major differences were identified, the agglomerated data are presented in this paper.

Coding was performed by a member of the research team (R-PF) using Microsoft Word and covalidated by another team member (AA), both trained by a researcher specialized in qualitative research (MC). Having interviewed all participants, AA was best suited to covalidate the codes, that is, to ensure that they reflected each case. The code list was then reviewed by MC, who was best suited to provide overall methodological feedback.

First cycle coding [31] is a way to initially summarize segments of data. As such, descriptive codes [31] were first assigned to all data in the interviews with HSCPs referring to *what* information about their client’s ADLs was relevant to them in the telemonitoring reports. Descriptive codes [31] were then assigned to all data in the interviews with HSCPs referring to *why* they deemed their client’s ADLs as adequate or not in regard to their home care. The descriptive coding process continued until data saturation was reached, meaning that descriptive codes had been assigned to all relevant elements [31] of the interviews with HSCPs, and no new codes emerged.

Pattern coding, as second cycle coding, focused mainly on causation, which is appropriate for discerning processes, as well as interrelationships and the complexity of influences on human actions [31]. Hence, pattern coding was used to connect first cycle descriptive codes (*what* and *why*) to understand *when* HSCPs made the clinical decision to modify their intervention (or not).

Once pattern coding was completed and data saturation reached again, flowcharts were drawn for each subcase to sequence the clinical decision-making process described in detail in the previous paragraphs (*what*, *why*, and *when*). All flowcharts belonging to HSCPs (ie, all subcases) within a social and health care institution (ie, 1 case) were then regrouped.

At this stage, preliminary analyses of the 3 flowcharts (CIUSSS 1, CIUSSS 2, and CISSS 3) augured similar results (ie, HSCPs used the telemonitoring reports in similar ways), which led to further analyses *within cases* (instead of across cases) [31] and thus 1 meta flowchart encompassing all 3 cases.

#### Quantitative Analysis

The quantitative data were analyzed on a per-person basis and, thereafter, all cases were combined to yield a comprehensive measure of the total monthly services received by the care recipients. Our aim was to investigate the impact of ADL telemonitoring reports on monthly service trends by comparing data collected before and after the introduction of the reports.

Initial analyses involved visually inspecting graphed data to identify specific trends [32]. Graphs depicted repeated outcomes before and after NEARS-SAPA implementation (respectively, the baseline and intervention time points), enabling a visual observation of trends between these 2 phases. Tau-*U* analysis [33], a statistical method commonly used in single-case experimental designs, was chosen to assess the stability, trend, and level of a dependent variable over time [34]. The Tau-*U* statistic is derived from the Kendall rank correlation and Mann-Whitney *U* tests. We selected the Tau-*U* due to its ability to account for large variability in the data, small sample size, nonparametric distribution, and trends in baseline and count-type data. All calculations were conducted using the Tau-*U* calculator website [35]. Specifically, Tau (no trend in the baseline) or Tau-*U* (a trend in the baseline) statistics were computed for each care recipient subcase and then aggregated by merging all nonoverlapping parameters to derive a global measure of nonoverlapping for the entire group of care recipients [33,36].

Given our relatively large sample size for Tau-*U* analyses, typically used in single-case designs, we also performed repeated ANOVAs for additional robustness. These analyses were conducted using SPSS (version 26.0; IBM Corp), with subcase transformed *z* scores used to mitigate the impact of large variability between subcases. Intervention (before vs after) and months (first to last) were entered as within-person factors.

Quantitative data were then embedded into the updated integration of the ambient assisted living monitoring technologies within clinical decision-making framework [15], which was supplemented by the qualitative data that emerged from this study.

## Results

### Study Samples

The 27 subcases, which included the care recipients and all HSCPs in charge of their care throughout the duration of the study, were distributed as follows: CIUSSS 1=5, 19%; CIUSSS 2=2, 7%; and CISSS 3=20, 74% (Table 1).

**Table 1:** Data collection details, including activity of daily living (ADL) telemonitoring reports delivered and follow-up interviews completed in each case.

			Cases				
			CIUSSS^a^ 1 (n=5), n (%)		CIUSSS 2 (n=2), n (%)		CISSS^b^ 3 (n=20), n (%)
Older adults (n=27)			5 (19)		2 (7)		20 (74)
**Health and social care professionals (n=34)**							
	Nurses	1 (7)		—^c^		1 (3)	
	Social workers	1 (7)		2 (7)		8 (28)	
	Occupational therapists	1 (3)		—		10 (34)	
	Physiotherapists	—		—		2 (7)	
	Respiratory therapists	—		—		1 (3)	
	Specialized educators	—		—		1 (3)	
ADL telemonitoring reports delivered (n=147)			39 (27)		6 (4)		102 (69)
Follow-up interviews (n=57)			7 (2)		4 (7)		46 (81)

^a^CIUSS: centre intégré universitaire de santé et de services sociaux (integrated university health and social services center).

^b^CISS: centre intégré de santé et de services sociaux (integrated health and social services center).

^c^Not applicable.

### HSCPs’ Characteristics

A total of 36 HSCPs were recruited to take part in the study. Of these 36 HSCPs, 7 professionals (CIUSSS1, n=3; CIUSSS2, n=2; CISSS3, n=2) were excluded from this analysis because postimplementation interviews could not be completed. The majority of the professionals were social workers (n=12, 41%), followed by occupational therapists (n=11, 38%), nurses (n=3, 10%), physiotherapists (n=2, 7%), and specialized educators (n=1, 3%). Of the 29 HSCPs, 6 followed more than one care recipients. In addition, 7 of the 27 care recipients were followed by 2 different HSCPs who were both interviewed. Overall, 93% (27/29) of HSCPs were women. The average age of the HSCPs was 41.50 (SD 9.29) years, and they had an average experience of 9.21 (SD 7.46) years. The number of postimplementation interviews completed by HSCPs varied according to the duration of their participation in the study. As such, 29 HSCPs completed a total of 57 postimplementation interviews, concerning 147 telemonitoring reports.

### Care Recipients’ Characteristics

While 31 care recipients were initially enrolled by HSCPs into the study, 4 (13%) were excluded later for lack of their HSCPs’ availability to complete postimplementation interviews. Thus, the ADL telemonitoring reports concerned 27 care recipients who took part in the study. Overall, 74% (20/27) of the care recipients were women. The average age of the care recipients was 81.30 (SD 7.67) years. Concerning Iso-SMAF profiles, 26% (7/27) of care recipients were categorized as having a “predominant loss in instrumental activities of daily life,” 11% (3/27) as having a “predominant loss in mobility functions,” 52% (14/27) as having a “predominant loss in cognitive functions,” and 11% (3/27) as having “serious mixed alterations.” Of the 27 care recipients, 16 (59%) completed the MMSE [29], with an average score of 20.94 (SD 5.49), while 9 (33%) completed the MoCA [30], with an average score of 19.33 (SD 5.79). All were older adults with cognitive deficits, and the vast majority (23/27, 85%) lived alone at home. Although living alone was one of our inclusion criteria, some care recipients did not live entirely alone but found themselves alone for long periods of time during the day. In such cases, it was possible to distinguish between the care recipient being alone and when a relative was also at home. Of the 27 care recipients, 2 (7%) were excluded from the quantitative ICLSC analyses because they did not consent to sharing their metadata.

More specifically, of the 5 care recipients from CIUSSS 1, the majority were women (n=3, 60%). The average age of these care recipients was 84.60 (SD 4.00; range 79-90) years. They had been diagnosed with Alzheimer disease (2/5, 40%), probable Alzheimer disease (1/5, 20%), or frontotemporal dementia (1/5, 20%). The MMSE [29] score was available for 3 (60%) of the 5 care recipients, with a mean of 23.00 (SD 6.00; range 16-28). The MoCA [30] score was also available for 3 (60%) of the 5 care recipients, with a mean of 23.00 (SD 6.00; range 17-29). Of the 5 care recipients, 1 (20%) had no cognitive diagnosis.

The 2 care recipients from CIUSSS 2 included 1 (50%) man and 1 (50%) woman, aged 79 and 88 years, respectively. Of these 2 care recipients, 1 (50%) was diagnosed with dementia due to a general medical condition, and 1 (50%) was diagnosed with an unspecified neurocognitive disorder. The MMSE [29] and MoCA [30] scores were available for 1 (50%) of these 2 care recipients and were 22 and 15, respectively.

Of the 20 care recipients from CISSS 3, the majority were women (n=16, 80%). The mean age of the care recipients was 80.00 (SD 8.00; range 65-90) years. They had been diagnosed with unspecified neurocognitive disorders (7/20, 35%), Alzheimer disease (6/20, 30%), probable Alzheimer disease (1/20, 5%), vascular dementia (1/20, 5%), or mixed dementia (3/20, 15%). Among those with mixed dementia, 33% (1/3) had major neurocognitive disorder combined with Korsakoff syndrome, 33% (1/3) had an unspecified neurocognitive disorder along with stroke, and 33% (1/3) had an unspecified diagnosis. The MMSE [29] score was available for 12 (60%) of the 20 care recipients, with a mean of 20.00 (SD 6.00; range 7-29). The MoCA [30] score was available for 5 (25%) of the 20 care recipients, with a mean of 18.00 (SD 6.00; range 11-24). Of the 20 care recipients, 1 (5%) had no cognitive diagnosis, while for 1 (5%), age and cognitive status were unavailable.

### Uses of NEARS-SAPA Telemonitoring Reports

#### Overview

**Figure 4:**
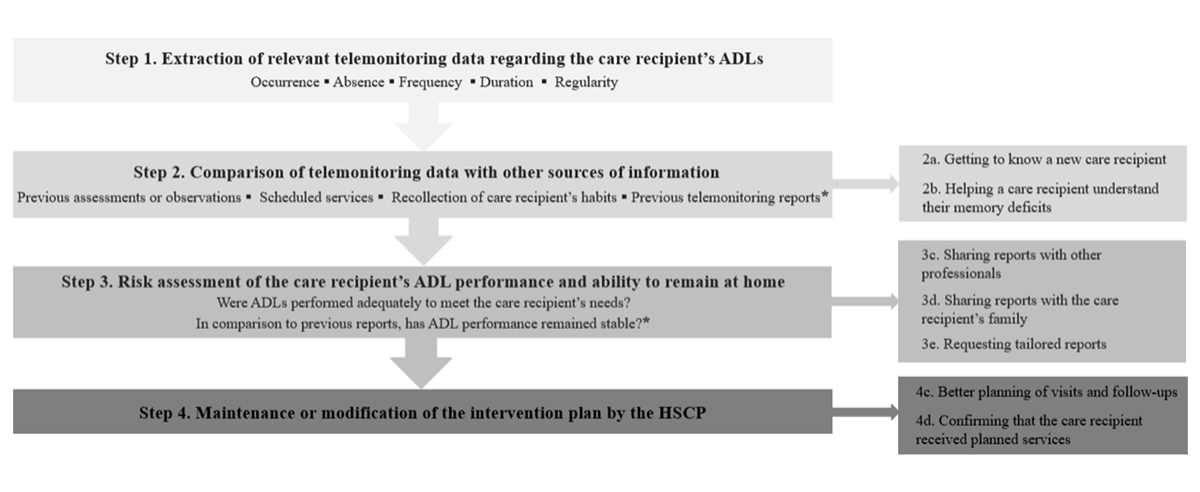
Uses of the Innovative Easy Assistance System–Support for Older Adults’ Autonomy program (Soutien à l’autonomie des personnes âgées in French; NEARS-SAPA) telemonitoring reports. ADL: activity of daily living; HSCP: health and social care professional. *Minor changes.

Figure 4 presents the most frequent uses of ADL telemonitoring reports in HSCPs’ decision-making concerning their care recipients’ home care situation, with particular regard to their needs (met or not) related to the 5 ADLs monitored by the NEARS-SAPA system (meal preparation and eating habits, personal hygiene, sleep, outings, and overall activity level).

The decision-making process comprised four main steps followed by most HSCPs: (1) extraction of relevant telemonitoring data, (2) comparison of telemonitoring data with other sources of information, (3) risk assessment of the care recipient’s ADL performance and ability to remain at home, and (4) maintenance or modification of the intervention plan. The boxes on the right-hand side in Figure 4 represent the additional, although less frequent, uses of telemonitoring reports at each corresponding step. Figure 4 also provides a more complete explanation of steps 3 and 4.

#### Step 1: Extraction of Relevant Telemonitoring Data Regarding the Care Recipients’ ADLs

Most HSCPs explained that, upon receiving their first telemonitoring report, they began by extracting relevant telemonitoring data, in particular the occurrence or absence, duration, frequency, and regularity of engagement in relevant ADLs by their care recipient.

The relevance of telemonitoring data varied among the HSCPs, depending on the needs of their care recipient, the challenges of their home care, and the clinical questions or concerns each HSCP had about their care recipient’s ADLs. Similarly, whether the relevant activities were deemed desirable (ie, aligned with their care recipient’s needs and supporting their home care) varied among the HSCPs; for example, using the oven might be considered a desirable activity for a care recipient who seldom prepared meals and only snacked on chips and cookies, whereas it might be considered undesirable for another care recipient who often left the oven unattended for a prolonged period and risked causing fires.

In the case of Judith (CIUSSS 2), the HSCP in charge worried that she did not eat appropriately or sufficiently outside of her afternoon and evening CLSC service hours. As such, the HSCP found the telemonitoring data about her activity in the kitchen especially relevant. The HSCP also considered the use of multiple kitchen appliances in the morning as a desirable activity:

In the morning, she has no services. So, we look to see if she’s using the coffee maker, the microwave, the toaster. It seems fine. She continues to use it. So, we can see that. Make sure she’s eating well.

HSCP in charge of Judith, CIUSSS 2

#### Step 2: Comparison of Telemonitoring Data With Other Sources of Information

Most HSCPs compared the data with other nontechnological sources of information about the care recipients’ ADLs, such as their own current and past observations or assessments of their abilities, as well as observations from family members, personal care assistants, or community organization staff during visits to their home. HSCPs also compared telemonitoring data with the care recipients’ self-reported activities or routines at home or their family’s account of these activities and routines.

Others compared telemonitoring data with services already in place to support care recipients, such as in the case of Roger (CISSS 3). After experiencing a stroke, Roger, who had neurocognitive deficits and physical difficulties, lived in a private residence for older adults. In Quebec, private older adult residences are rental buildings occupied or intended to be occupied primarily by older adults aged ≥65 years and where various services are offered, such as meals, housekeeping, nursing care, and leisure activities [37]. Although Roger did not have problematic eating habits, the HSCP in charge of his care still wanted information about his overall routine. The telemonitoring report showed that Roger left his apartment 3 times a day, always at the same time. The HSCP compared the data with the scheduled meals at the older adult residence:

Also, I looked at the outings. The fact that he goes out three times a day at the same times, which corresponds to the meals he gets.

HSCP in charge of Roger, CISSS 3

Some HSCPs also combined telemonitoring reports with their care recipient’s recollections of their daily habits, such as in the case of Chantale (CISSS 3; step 2b). Chantale had personal hygiene problems, and her family worried about this; however, she claimed to shower daily. The telemonitoring report showed no activity in the shower. The HSCP in charge explained how telemonitoring data can help in such situations:

It also enables us, in our interventions with the lady, not to confront her, but to say to her ‘well, what we have as data concerning you is not what you’re telling us.’ So it gives us a kind of leverage to make her understand that maybe her memory is playing tricks on her too.

HSCP in charge of Chantale, CISSS 3

#### Step 3: Risk Assessment of Care Recipients’ ADL Performance

##### Overview

**Figure 5:**
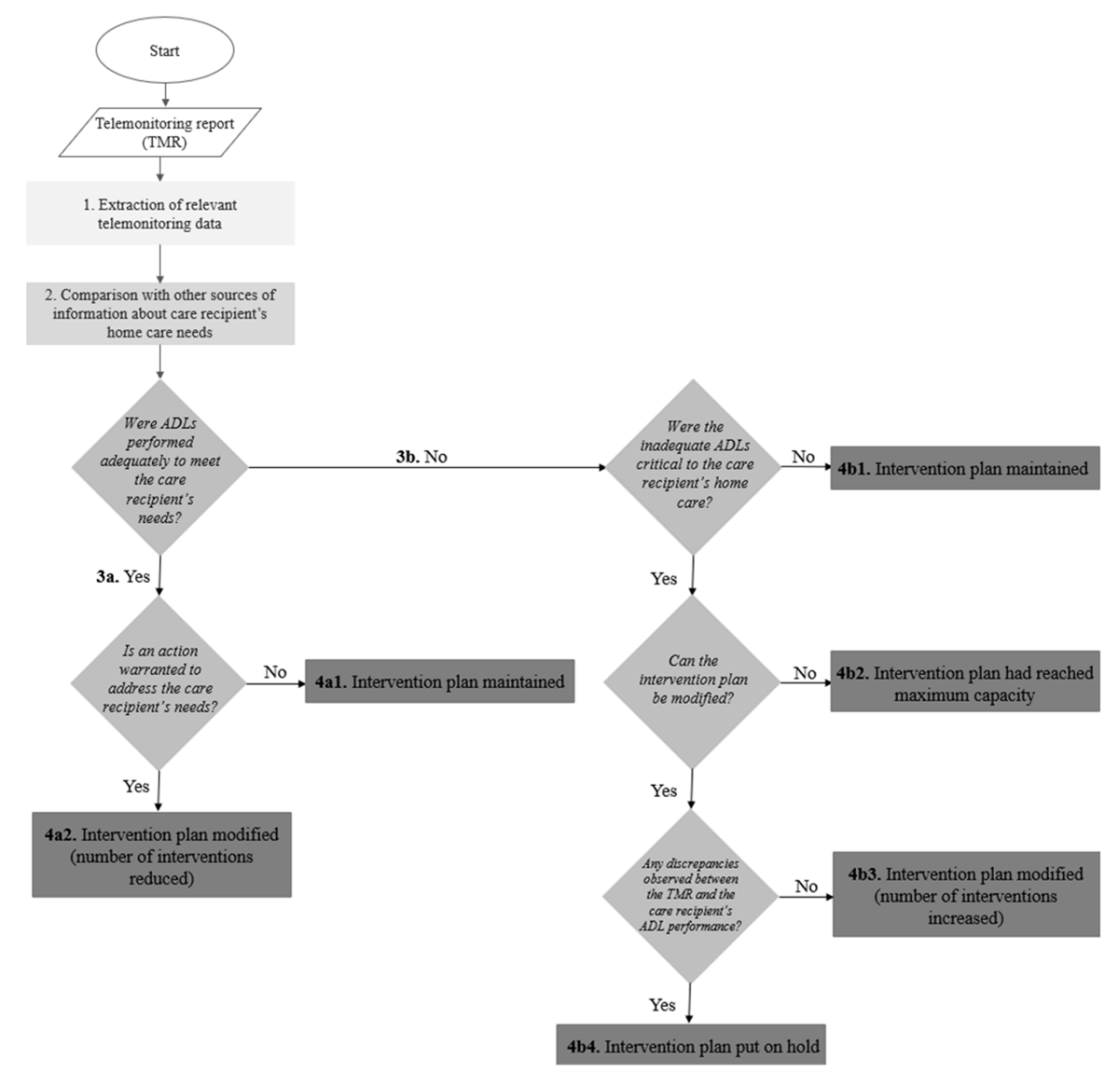
Impact of the telemonitoring report on risk assessment and service delivery. ADL: activity of daily living.

After comparing the telemonitoring data with other sources of information, HSCPs proceeded with the risk assessment of their care recipient’s ADL performance for their home care (step 3). Substeps 3a and 3b are detailed in Figure 5, and examples are presented in Table 2.

**Table 2:** Codes, definitions, and verbatim extracts of interviews with health and social care professionals (HSCPs) for step 3: risk assessment of the performance of activities of daily living (ADLs).

Codes	Definitions	Context	Verbatim interview extracts
Substep 3a. Care recipient’s ADL performance is adequate	Most HSCPs deemed ADL performance to be adequate when the care recipient performed activities adequately to meet their home care needs (ie, in terms of frequency, regularity, occurrence or absence, etc).	Serge lived at home with his wife, and both had neurocognitive deficits. When the HSCP in charge of their care initially joined the research project, Serge still had his car and would leave home in the middle of the night. Although he eventually stopped using his car, the HSCP wanted to know whether he still went out at night. The telemonitoring report showed that Serge did not go out at night.	“[W]hen I made the request [to be part of the study], he still had his car. So, he was going out at night...There were nocturnal outings, too, that worried us when he was there. So, it was fun to see on the sensors that he wasn’t going out, you know.” [HSCP in charge of Serge, CISSS^a^ 3]
Substep 3b. Care recipient’s ADL performance is inadequate	Most HSCPs deemed ADL performance to be inadequate when the care recipient did not perform activities adequately to meet their home care needs (ie, in terms of frequency, regularity, occurrence or absence, etc).	Francine, an older woman recently diagnosed with neurocognitive deficits, had a tendency to minimize her difficulties. While she reported no sleep issues, her son reported that she was agitated during the night, a concern not shared by her husband. The telemonitoring report shed light on these contradictory accounts: it showed that Francine did sometimes wake up and become active at night.	“We noticed that, at night, she tended to get up and sleep in blocks. So, it confirmed what the son had told us, that at night she doesn’t necessarily sleep like everyone else. Let’s say, she goes to bed and then gets up the next morning: there are also periods when she’s more active, so it confirmed that aspect.” [HSCP in charge of Francine, CISSS 3]
Substep 3c. Share information with other professionals	Some HSCPs shared the telemonitoring reports with other professionals involved in their client’s home care, whether their ADL performance was adequate or not.	Bonnie had neurocognitive deficits and had twice put her life in danger by leaving her home without a coat on. Outings were the main obstacle to her home care. The HSCP in charge of her care was concerned because her surroundings, including stairways and backyard, were very cluttered, and she lived close to a river. The telemonitoring report showed that the outings Bonnie went on did not last long.	“It gave a lot of interesting information. It allows you to objectify what you think. You know, by deduction and then with the clinical discussions that you have as a team. But it allows you to objectify. That was really, really appreciated by our team.” [HSCP in charge of Bonnie, CISSS 3]
Substep 3d. Share information with client’s family to reassure them	Some HSCPs shared the telemonitoring report with their client’s family when it showed that the care recipient performed ADLs adequately (eg, regular and desirable activities) to reassure them about their home care.	Marie, who lived alone, had neurocognitive deficits, and her daughter was very worried about the precarity of her home care, especially in regard to her outings and eating habits. The telemonitoring reports showed that the outings were minimal, of short duration, and never at night and that Marie was active in the kitchen even outside of CLSC^b^ service hours.	“Actually, that’s it, she was reassured. She worried that her mother would leave for longer periods of time or maybe a little more frequently eventually. Which is not the case. There was also, if I may add a little bit, concerning the meals, it allows us to see that the lady seems to eat even outside of CLSC service hours, at least to take snacks and all that. Which is reassuring for the daughter because she feared that Madame did not eat or barely did, you know.” [HSCP in charge of Marie, CISSS 3]
Substep 3d. Share information with client’s family to support relocation	Some HSCPs shared the telemonitoring report with their client’s family when it showed that the care recipient did not perform ADLs adequately (eg, prolonged, undesirable activities) to support their decision to relocate their family member.	Maggie had severe cognitive difficulties, and her home care was known by the HSCP in charge of her care to be precarious; yet, her daughter worried that relocation would deprive her of her quality of life. The telemonitoring reports showed that, when she was alone at home, Maggie spent on average close to 14.5 hours per day in bed.	“It’s mostly with sleep. Because, you know, she spends an average of 14-and-a-half hours a day in her room. In her bed. That’s a lot of sleep. And during the day too. With little stimulation...sleep, in fact, it helped her to put on, well, not weight, but to help her make the decision, for relocation, because, on the one hand, she [Maggie’s daughter] was concerned about quality of life and taking something away from her by moving towards relocation, but on the other hand, if you look, well, she sleeps...She spent a lot of time in bed anyway, and things like that, so we could still rely on that...so, you know, it also helped her to better understand reality.” [HSCP in charge of Maggie, CIUSSS^c^ 1]

^a^CISSS: centre intégré de santé et de services sociaux (integrated health and social services center).

^b^CLSC: centre locaux de services communautaires (local community service center).

^c^CIUSSS: centre intégré universitaire de santé et de services sociaux (integrated university health and social services center).

##### ADLs Performed Adequately (Step 3a)

ADLs were considered as adequately performed when the telemonitoring report showed that the care recipient (1) engaged in desirable activities regularly (eg, “the user ate their meals at regular hours on most days”), (2) engaged in desirable activities frequently (eg, “the user showered every day”), or (3) avoided potentially dangerous or undesirable activities.

##### ADLs Performed Inadequately (Step 3b)

ADLs were considered as inadequately performed when the telemonitoring reports showed that the care recipient (1) engaged in undesirable activities frequently (eg, “the person frequently wanders out at night”), (2) engaged in undesirable activities for a prolonged period (eg, “the person spends more than 13 hours a day in bed”), (3) performed activities less frequently than before (eg, “the user does not go out as much as they used to”), (4) performed activities scarcely, or (5) performed activities for an insufficient duration (eg, “the person barely sleeps at night”).

##### Sharing the Data With Others (Steps 3c and 3d)

Whether ADL performance was adequate or not, some HSCPs reported that the telemonitoring data supported their risk assessment of their care recipient’s home care by allowing them to share information with other professionals, such as in the case of Bonnie (Table 2). Moreover, some HSCPs used the telemonitoring reports to discuss their risk assessment with the family, such as in the cases of Marie and Maggie (Table 2). In both Marie’s and Maggie’s cases, HSCPs shared the telemonitoring reports with their care recipients’ daughters, although this led to very different outcomes. In Marie’s case, being informed of her mother’s adequate eating habits reassured the daughter about her home care situation. By contrast, being informed of Maggie’s inadequate sleeping habits supported her daughter’s decision to relocate her because, based on the telemonitoring data, relocation would not deprive her of her quality of life as she spent more than half the day on most days in bed and thus received little stimulation at home.

##### Need for Tailored Telemonitoring Reports (Step 3e)

When specific questions remained unanswered after receiving a telemonitoring report, some HSCPs made requests to the research team for more tailored telemonitoring reports to enable them to make an accurate risk assessment. As such, tailored telemonitoring reports differed from the more generic reports in that they were more granular and targeted specific actions, specific days, and specific sensors; for example, Esther (CISSS 3), who lived alone, had neurocognitive deficits, and found it difficult to manage her diabetes. She had experienced a severe hypoglycemic episode in the past, which had led the HSCP in charge of her care to implement a new strategy to have her measure her blood glucose level after dinner and eat a snack:

That’s why we make her take it [her blood glucose level] in the evening. Basically, at suppertime, the auxiliary tells her, “Tonight, you take your blood sugar.” She takes it, and then she has her snack. That’s why we started this program, to make up for that [past severe hypoglycemic episode].

HSCP in charge of Esther, CISSS 3

Nevertheless, after the implementation of the strategy by the HSCP, more hypoglycemic episodes occurred. The HSCP doubted whether Esther took her evening snack as directed and requested a tailored telemonitoring report specifically on evening snack consumption. The report showed that, contrary to her claim, Esther did not systematically eat her evening snack:

So, what I did is that I asked [the research professional] to get the data out. We realized that it was more like three times out of four...So, I know that three days out of four she takes it, then one day out of four she doesn’t, so Mrs...I can see the discrepancy with what Mrs tells me.

HSCP in charge of Esther, CISSS 3

#### Step 4: Maintenance or Modification of the Intervention Plan

##### Overview

After assessing the risks for their care recipients, HSCPs made decisions regarding their intervention plan (step 4). The most frequent decision reported by HSCPs in interviews was to maintain the actual intervention plan (43 occurrences recorded). The other decisions reported were to increase the number of interventions (2 occurrences) and to reduce the number of interventions (1 occurrence). Multimedia Appendix 1 presents details per type of ADL. Step 4 substeps are detailed in Figures 4 and 5, and examples are presented in Multimedia Appendix 1.

##### ADLs Performed Adequately, Whether Critical or Noncritical (Steps 4a1 and 4a2)

When care recipients performed ADLs adequately to meet their needs, most HSCPs deemed it unnecessary to implement a new intervention or modify their intervention plan (Multimedia Appendix 1). In some cases, the telemonitoring reports also supported the reduction in the number of interventions (Multimedia Appendix 1).

##### Noncritical ADLs Performed Inadequately (Step 4b1)

When the telemonitoring reports showed that the care recipients were performing noncritical ADLs inadequately, most HSCPs deemed it unnecessary to implement a new intervention or modify their intervention plan (Multimedia Appendix 1).

##### Critical ADLs Performed Inadequately (Steps 4b2-4b4)

Some HSCPs faced with a critical home care situation were not able to take action to address their care recipients’ inadequate activities, such as in the case of Steve (Multimedia Appendix 1). Indeed, the HSCP concluded that once Steve could no longer perform the “bare minimum” of ADLs, relocation would be the next step because their intervention plan had already reached maximum capacity. Some HSCPs increased the number of interventions offered (Multimedia Appendix 1). Finally, when discrepancies arose between the telemonitoring reports and other sources of information about the care recipients’ critical ADL performance, HSCPs often opted to put their intervention plan “on hold” to carry out an investigation. The term “on hold” was used when the HSCP and the research team required some time to investigate discrepancies between their observations and the telemonitoring data to decide whether a modification to the intervention plan was indeed warranted. As such, the actual plan was maintained but subject to future changes. The “on hold” cases were different from cases in which the intervention plan was maintained because ADLs were performed adequately and ADLs performed inadequately were noncritical to the care recipient’s home care. Cases were put “on hold” when the reports showed that the care recipients scarcely performed desirable activities for which scheduled services were already in place (Multimedia Appendix 1) or when they showed the care recipients performing desirable activities that were inconsistent with the HSCP’s observations (Multimedia Appendix 1). In Lois’s and Louise’s cases, discrepant telemonitoring data led the HSCP to inquire about sensor locations in the kitchen and bathroom, respectively. These inquiries with the research team led to improved sensor location, which in turn allowed for more accurate detection of the women’s activities as well as more accurate subsequent telemonitoring reports.

##### Timing of Interventions (Step 4c)

Some HSCPs used the telemonitoring reports to improve the timing of their interventions, notably by planning visits and follow-ups at the moments when the reports showed their care recipients to regularly be at home and active (Multimedia Appendix 1).

##### Confirming the Delivery of the Intervention Plan (Step 4d)

HSCPs also used the telemonitoring reports to confirm that their intervention plan was being followed, notably by checking for increased activity in the care recipient’s home during planned service hours, with home care delivered by health and social services assistants (Multimedia Appendix 1).

### Subsequent Telemonitoring Reports: Assessment of ADL Performance Stability

With each subsequent telemonitoring report (refer to Table 3 for examples), HSCPs went through the same clinical decision-making steps illustrated in Figure 4, with minor changes (identified with an asterisk). Indeed, after thoroughly combining the contents of the first telemonitoring report with other sources of information about their care recipient’s ADLs (eg, their own observations), HSCPs considered telemonitoring reports to be a valid (ie, accurate and representative) source of *additional* information. From then on, they compared the telemonitoring reports with *previous telemonitoring reports* (step 2). As such, they assessed the risk of maintaining the care recipient at home (step 3) by asking themselves whether, *in comparison to previous reports, the care recipient’s ADL routine was stable.* Hence, most HSCPs deemed that a modification to their intervention plan was unnecessary if their care recipient’s ADL performance had remained stable since the last telemonitoring report.

By contrast, a modification to the intervention plan was warranted if the care recipients’ ADL routine was unstable and thus reflected a deterioration (eg, in comparison to the previous telemonitoring report, the care recipient now barely showers) or if undesirable activities had not improved or had worsened since then (eg, in comparison to the previous telemonitoring report, the care recipient now regularly wanders at night).

**Table 3:** Codes, definitions, and verbatim extracts of interviews with health and social care professionals (HSCPs) for subsequent telemonitoring reports: assessment of activity of daily living (ADL) performance stability.

Care recipient and codes			Definitions		Context		Verbatim interview extracts
Donna **(CISSS**^a^ **3)**							
	First report: maintenance of the intervention plan	Some HSCPs maintained their intervention plan when the telemonitoring report showed that the care recipient performed ADLs adequately to meet their needs (eg, they did not perform undesirable activities).		While Donna’s cognitive difficulties made it difficult to explain her situation to the building managers, the first telemonitoring report showed that there was barely any activity at night and that Donna remained in her bedroom, thus disproving the neighbor’s claims (refer to the corresponding interview extract). This allowed the HSCP in charge of her care to provide the building managers with information before they began the eviction process with the Quebec Regie du logement (Quebec Administrative Housing Tribunal), as they planned.		“And when I told them [the building managers] that, well, Madame had sensors in her home and everything, that we could see what was going on at night and that what it showed us was that there wasn’t really any activity going on. She’s in her room most of the time, whereas the complaints were that she was in her bathroom and making a lot of noise in there...Anyway, anyway! I explained everything and it seems that the situation is back to normal. They’ve offered her a new lease. You know, I don’t have all the final, final details, but we’ll be there. Madame, she’s got a lease. She just signed a lease for a year.” [HSCP in charge of Donna, CISSS 3]	
	Subsequent report: maintenance of the intervention plan	Some HSCPs maintained their intervention plan when the subsequent telemonitoring report still showed that the care recipient performed ADLs adequately to meet their needs (eg, they still did not perform undesirable activities).		The subsequent telemonitoring report showed the stability of Donna’s routine. The HSCP used this report to make the clinical decision that there still was no need for an intervention (or relocation) to address her home care situation. Donna was offered a new lease, and her relocation was no longer deemed necessary.		“I still get reports from [the research professional], and it’s pretty stable. What I understand is that her routine is stable. She doesn’t leave her apartment much, she doesn’t have too many visitors, the door doesn’t open that much...Despite everything she went through, she eats, she washes, she has a routine. You see, she goes to bed at relatively the same time, she gets up at the same time.” [HSCP in charge of Donna, CISSS 3]	
Rose **(CISSS 3)**							
	First report: intervention plan put on hold	Some HSCPs put their intervention plan on hold when the telemonitoring report showed that the care recipient did not perform ADLs adequately to meet their needs (eg, they frequently performed undesirable activities), which the family doubted was accurate.		Rose lived alone in a basement apartment in her daughter’s home. Upon receiving the first telemonitoring report, questions arose for both Rose’s daughter and the HSCP: it showed that Rose frequently woke up during the night and went upstairs. The HSCP initially put their intervention plan on hold to assess the possibility with the research team that the sensors were in fact detecting the family dog, not Rose.		“So, the daughter doesn’t think that her mother goes upstairs to her apartment in the middle of the night. She says, “She sleeps well.” But she says, “The dog, it’s more likely that it was the dog that had gone upstairs.” So, it would be a matter of validating it. Because I know he [the research professional] was very careful to put them [the sensors] on precisely so that it wouldn’t detect the dog.” [HSCP in charge of Rose, CISSS 3]	
	Subsequent report: addition to the intervention plan	Some HSCPs added an intervention to their intervention plan when the subsequent report still showed that the care recipient did not perform ADLs adequately to meet their needs (eg, they still frequently performed undesirable activities).		When the HSCP received the subsequent telemonitoring report—which still showed Rose going upstairs during the night—follow-ups were initiated with her daughter to better secure her medication, which was kept upstairs.		“In what way did it influence...well, basically it allowed...well, because of her comings and goings, and we could see that she was going upstairs. Above all, it influenced the follow-up with her daughter to put in place safer measures for her medication.” [HSCP in charge of Rose, CISSS 3]	

^a^CISSS: *centre intégré de santé et de services sociaux* (integrated health and social services center).

### Quantitative Indicators of the Impact of ADL Telemonitoring Reports on the Intervention Plan

**Figure 6:**
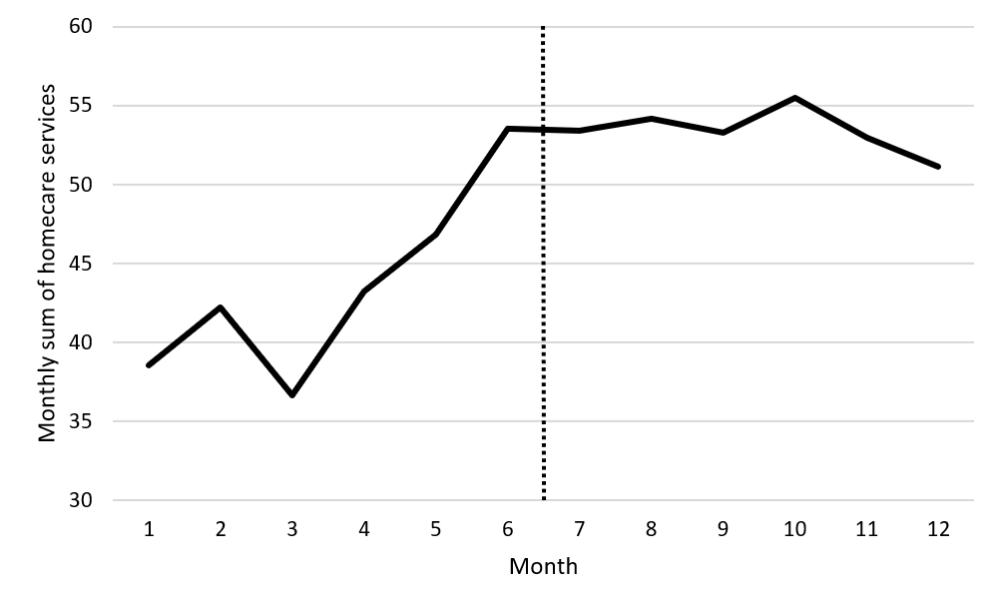
Total monthly home care services received over time before the introduction of Innovative Easy Assistance System–Support for Older Adults’ Autonomy program (Soutien à l’autonomie des personnes âgées in French; NEARS-SAPA).

**Figure 7:**
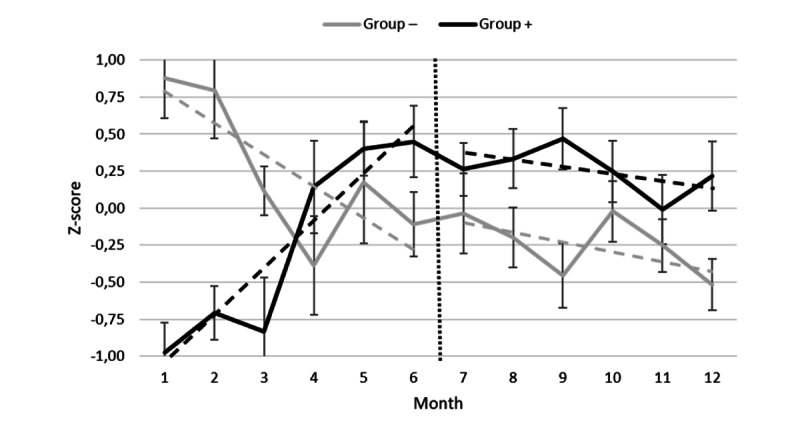
Visual analysis of subcases showing 2 distinct profiles, that is, group− and group+.

The quantitative data complement the qualitative information obtained from the HSCPs describing how they maintain or modify their intervention plan according to the ADL telemonitoring report (ie, step 4 and subsequent telemonitoring report use). We initially conducted a visual analysis of graphed data for each case to discern specific trends in services provided in correlation with NEARS-SAPA reports. Initial examination of the overall data suggests a gradual increase in the total monthly home care services received over time before the introduction of NEARS-SAPA (Figure 6), with a decrease in added services after its implementation. However, Tau-*U* analyses revealed no significant (Tau-*U*=0.14; *P*=.09) changes in trends from before to after the intervention. However, scrutinizing individual participant curves revealed 2 distinct patterns, which obscured outcomes when merged together. When solely considering the period before the introduction of NEARS-SAPA, 2 separate baseline trends emerged (Figure 7): (1) those in which the number of services provided remained stable or decreased before the intervention (group−; Tau ≤0, n=12), and (2) those in which the number of services provided increased before the intervention (group+; Tau >0, n=13). Subsequent analyses were conducted separately for these 2 groups to avoid conflicting trends from overlapping in the same analysis. Both groups were comparable in terms of age (t_23_=−1.56; *P*=.13), sex (t_23_=−0.10; *P*=.91), and Iso-SMAF profiles (t_23_=−0.87; *P*=.53).

In the case of group+, Tau-*U* analyses showed a significant change of medium effect size in trend after the introduction of NEARS-SAPA (Tau-*U*=−0.25; *P*=.009). This finding aligns with the outcomes of repeated ANOVAs: a significant intervention×months interaction (*F*_1,12_=7.34; *P*<.001; η^2^=0.38) revealed notable differences in trends before and after the introduction of NEARS-SAPA. Before the introduction of NEARS-SAPA, there was a substantial increase in services provided over time (Tau-*U*=0.52; *P*<.001). However, after the introduction of NEARS-SAPA, there was no significant increase in services during subsequent months (Tau-*U*=−0.01; *P*=.94). In essence, this indicates a stabilization in monthly services after the implementation of NEARS-SAPA.

The Tau-*U* analysis conducted for group− also exhibited a noteworthy change in trend subsequent to the introduction of NEARS-SAPA, as evidenced by a medium effect size (Tau-*U*=−0.27; *P*=.008). Upon closer examination, we noted a moderate decrease in services provided before the introduction of NEARS-SAPA (Tau-*U*=−0.31; *P*=.002), whereas this reduction was no longer significant after the introduction of NEARS-SAPA (Tau-*U*=−0.12; *P*=.25). However, this finding did not hold under repeated ANOVAs because the differences in service decreases before and after the introduction of NEARS-SAPA were not statistically significant.

Overall, the results suggest a stabilization in monthly services received after the introduction of NEARS-SAPA compared to the 6 months prior. This effect was more significant in cases where services were increasing before its implementation.

## Discussion

### Principal Findings

We conducted an ADR project to codevelop and implement NEARS-SAPA, an ADL telemonitoring system aiming to support home care services for care recipients with cognitive deficits. As part of this larger ADR project, the objectives of this study were more specifically to describe (1) how the ADL telemonitoring data were used by HSCPs in the process of maintaining care recipients with cognitive deficits at home, and (2) the impact of ADL telemonitoring on service delivery. For objective 2, we hypothesized that ADL telemonitoring would contribute to service optimization, in that it would enable HSCPs to better identify which type of services their care recipient would need throughout the evolution of their condition.

To our knowledge, this is the first study to report on the use of ADL telemonitoring in real health care services and, in particular, in the decision-making process of HSCPs [12]. With regard to objective 1, the HSCPs reported their use of the ADL telemonitoring data in the form of a series of steps aiming to assess the suitability of the existing intervention plan. More specifically, once the telemonitoring data were extracted and compared with other relevant sources of information, HSCPs conducted a risk assessment of the care recipient’s ADL performance and ability to remain at home and then acted on their intervention plan. With regard to objective 2, HSCPs reported that their intervention plan could be maintained, modified (number of interventions reduced or increased), or put on hold. Care recipients’ clinical metadata showed a stabilization in services received after the introduction of NEARS-SAPA, especially in cases where services were increasing before its implementation. This is consistent with qualitative data indicating that, in light of the telemonitoring data, most HSCPs decided to maintain the existing intervention plan rather than increasing or reducing services. Consequently, we observed fewer significant changes in home care services after the introduction of ADL telemonitoring. Still, interpretation must remain prudent because the distribution of services is influenced by multiple factors, and the results need to be replicated with a larger sample size and a control group. Overall, the qualitative results confirm our hypothesis that ADL telemonitoring contributed to service optimization on a case-by-case basis. These preliminary quantitative results suggest that ADL telemonitoring has the potential to influence service delivery on a larger scale, in particular when questions remain regarding the relevance of services. ADL telemonitoring seems to have the potential to play an important role in reassuring HSCPs about their risk management and the appropriateness of service delivery. NEARS-SAPA enabled the clinicians to obtain information that was previously unavailable, contradictory, or only partially accessible.

For home care workers, risk management is central to their practice, particularly for the home care of care recipients with cognitive deficits. Professionals and caregivers who care for them define this risk management as fluid and highly context dependent [38], thus encompassing subjective aspects [39]. In a study conducted among home care staff in Sweden, Sandberg et al [40] reported that these workers implement a reasoning process aimed at tracking, identifying, and acting on this risk. Tracking and identifying risks were considered challenging by the participants, requiring close and longitudinal monitoring because needs evolve with the progression of cognitive impairments; they described how they were constantly on the lookout for signs of risk during home visits, which can occur several times a day. In our study, HSCPs reported that ADL telemonitoring has the potential to reduce certain subjective aspects related to risk management; facilitate longitudinal monitoring; and, in some contexts, reduce the need to travel to confirm certain information.

However, ADL telemonitoring only provides a few pieces of the puzzle. There are several factors contributing to the risks at home and the maintenance of the individual in their living environment, factors that cannot be assessed through telemonitoring, such as each HSCP’s comfort level with risk, ethical dilemmas related to the protection of the individual versus their expressed needs and wishes, caregiver level of burden, the care recipient’s socioeconomic status, complex medication management, and so on [23,40,41]. The decision to add a service or recommend a change in living environments to avoid excessive risks thus relies on a multitude of factors, and it is possible that ADL telemonitoring does not have a direct effect on the services received. This is why the quantitative data in this study may not, at this stage at least, show significant direct changes in all contexts. Interpretation must remain prudent because the distribution of services is influenced by multiple factors, and the causality between the introduction of telemonitoring and the stabilization of services has not been established in this study. To strengthen these results, future research should replicate the findings with a larger sample size and include a control group matched to the experimental group on key factors such as age, health conditions, duration since first receiving home care services, and time since the last functional evaluation. Future larger-scale studies with control groups may be able to identify more specific effects of ADL telemonitoring on service delivery and associated costs.

The results of this study also suggest that the NEARS-SAPA system may be considered akin to a clinical decision support system. According to Sutton et al [42], “a clinical decision support system...is intended to improve healthcare delivery by enhancing medical decisions with targeted clinical knowledge, patient information, and other health information.” However, NEARS-SAPA does not issue recommendations on the decisions and actions to be taken and therefore does not perfectly fit with the classical notion of a clinical decision support system [42]. Lyell et al [43] suggest the use of the term “assistive technology” for clinical decision-making to refer to those types of systems where part of the data extraction and analysis is done by algorithms but the clinicians provide the decision regarding the task and the need to confirm or approve the information provided by the system. This distinction is important when one seeks to compare the results of our study with those of other types of telemonitoring systems. The use of data differs between these contexts: in some systems, clinicians use a recommendation indicating changes in a patient’s health status that require follow-up (eg, oxygen saturation and heart rate indicate deterioration from the previous day or from hospital discharge), whereas in the case of NEARS-SAPA, HSCPs used uninterpreted evaluation results (eg, care recipient showers once a week). Future studies could explore the potential challenges and limitations faced by clinicians when integrating ADL telemonitoring data into their decision-making process to provide a complete view of the implementation processes of such systems.

That said, despite the distinction between the 2 types of systems, there are no substantial data on how clinicians use clinical decision support systems or assistive technologies in their clinical reasoning, particularly in real clinical practice contexts [43]. Barken et al [44] conducted one of the few studies, to our knowledge, that have examined how the telemonitoring of parameters related to chronic diseases is integrated into the clinical decision-making of nurses dedicated to this task. The authors showed that nurses combine various pieces of information, including telemonitoring, to obtain an overall view of the patient’s condition and clarify their current health status. Similar to the HSCPs in this study, the nurses in the study by Barken et al [44] used telemonitoring as a complementary source of information. They use it to initiate their clinical reasoning, that is, to identify health problems and then independently prioritize the patients’ follow-up needs. In our study’s context, the information was specifically used to address questions about potential risks in a setting where clinical reasoning had already begun and where the HSCP was interested in obtaining additional or supplementary information. It would be interesting to study in the future whether the framework that emerged from our study could apply to the use of other types of telemonitoring, such as those related to chronic diseases, as well as in other clinical contexts (eg, when clinical reasoning is in its infancy, such as for new care recipients). Understanding the clinical reasoning of professionals using telemonitoring is crucial to help field teams prepare for the deployment of such systems and integrate them harmoniously with existing practices [45]. This careful planning of integration can positively impact the adoption and sustainable maintenance of the technology, knowing that many technology deployment projects in health care have failed worldwide [45]. Understanding how technology can truly be useful to health care workers is an important consideration.

### Limitations and Strengths

The work presented here is a first step, but we are aware that our work still has limited scope. This limitation is also partly due to the COVID-19 context, which forced us to use manually created PDF reports to present the data and help HSCPs understand the significance of the data. This made the process laborious and difficult to scale up. Future studies will focus particularly on ensuring automated and clear data presentation so that the system can be more easily used on a larger scale while continuing to effectively support clinical reasoning. The limited scope of our study is also related to the absence of a control group, which particularly impacted the analysis of quantitative results. In addition, the scope is limited by the fact that the older adults followed in our study were mostly already known to home care services and were already receiving services. Thus, we do not know how ADL telemonitoring could be used in the context of an initial contact with a user for a first needs assessment. The use of ADL telemonitoring data could be different at this stage and have more cost savings related to the time saved in obtaining the initial clinical information required to develop an intervention plan. In addition, we did not document the cost impacts related to service delivery and the benefits for the health care system in a more holistic manner. Finally, another important point to address in the future is the ethical issues that can arise during the implementation of a system such as NEARS-SAPA. These ethical aspects should be explored in greater depth with all stakeholders to ensure that all potential ethical issues are considered a priori and during the course of the study. Future larger-scale studies will allow us to analyze both cost and ethical aspects.

However, one of the great strengths of our study is the fact that it relied on mixed longitudinal data and a large number of interviews, enabling a rigorous and innovative investigation of the use of ADL telemonitoring data in a real clinical setting.

### Conclusions

To our knowledge, this is the first demonstration of the integration of ADL telemonitoring data in a real clinical practice setting. It is also the first description of the real potential impact of this technology on the delivery of home support services. These results will help future studies and health care managers better target the role of new technologies in home care clinical practice. Future studies may further explore the benefits of ADL telemonitoring (qualitative and quantitative) for public health care systems, with larger-scale implementation studies.

## Appendix

Multimedia Appendix 1 Codes, definitions, and verbatim extracts of interviews with health and social care professionals for step 4: maintenance or modification of the intervention plan.

## Abbreviations

ADL: activity of daily living
ADR: action design research
CISSS: centre intégré de santé et de services sociaux (integrated health and social services center)
CIUSSS: centre intégré universitaire de santé et de services sociaux (integrated university health and social services center)
CLSC: centres locaux de services communautaires (local community service center)
COREQ: Consolidated Criteria for Reporting Qualitative Research
HSCP: health and social care professional
Iso-SMAF: Iso-Système de mesure de l’autonomie fonctionnelle
MMSE: Mini-Mental State Examination
MoCA: Montreal Cognitive Assessment
NEARS: Innovative Easy Assistance System
SAPA: Soutien à l’autonomie des personnes âgées (Support for Older Adults’ Autonomy program)
