# Supplementary material for: Decision-Making Process of Home and Social Care Professionals Using Telemonitoring of Activities of Daily Living for Risk Assessment: Embedded Mixed Methods Multiple-Case Study
Source: J Med Internet Res. 2025 Apr 25;27:e64713. doi: 10.2196/64713 (PMC12064971; doi:10.2196/64713)
Supplement: Multimedia Appendix 1 [file jmir_v27i1e64713_app1.docx]

Multimedia Appendix 1. Codes, definitions, and verbatim extracts of interviews with health and social care professionals (HSCPs) for step 4: maintenance or modification of the intervention plan.

| Codes | Definitions | Context | Verbatim interview extracts |
| --- | --- | --- | --- |
| Substep 4a1. Maintenance of the intervention plan | - Most HSCPs deemed it unnecessary to add an intervention when the telemonitoring report showed that the care recipient performed ADLs adequately (eg, they did not perform potentially dangerous or undesirable activities). - Occurrences of ADLs recorded: meal preparation=18, sleep=7, hygiene=8, outings=9, and activity level=1. | - In the case of *Marge*, who lived alone and had neurocognitive difficulties, the HSCP was concerned about her safety after she had reported turning on the oven and leaving its door open when she felt cold. In addition, in a prior meal preparation assessment with the HSCP, *Marge* had forgotten to turn off the oven. Hence, adapting the oven for her safety was a potential intervention the HSCP was considering. However, when the telemonitoring report showed that *Marge* did not use the oven for prolonged periods, its use was deemed safe by the HSCP and this intervention was not implemented. | - “And it also allowed me to, you know, in terms of stove use, I had doubts about the validity of my assessment in the sense that the wife had forgotten to turn it off...So, I was happy to see that the stove wasn’t left on for worrying lengths of time versus, in my assessment, she’d forgotten to turn it off...Well, we didn’t adapt the stove after all. The fact is, she was able to keep her habits, which was even better for her than upsetting her...she functions very much by automatism, so, like, I was afraid of introducing a new adaptation. So, we didn’t have to upset her daily routine.” [HSCP in charge of *Marge*, CISSS 3] |
| Substep 4a2. Reduction of the intervention plan | - Some HSCPs reduced an intervention when the telemonitoring report showed that the care recipient performed ADLs adequately (eg, they performed desirable activities regularly). - Occurrences of ADLs recorded: meal preparation=1, sleep=0, hygiene=0, outings=0, and activity level=0. | - After experiencing a stroke, *Thérèse* had been admitted to hospital, where she had been under careful supervision by nurses (especially for medication). Some time after returning home, *Thérèse* wanted more autonomy. While the HSCP in charge of her care believed that too many services were now in place, her family remained greatly concerned. The telemonitoring report showed that *Thérèse* had good eating habits and that she used the microwave mostly around lunchtime and the oven mostly in the evening. The telemonitoring report also showed that *Thérèse* was active in the kitchen at regular times to prepare meals. - *Stan* had Alzheimer disease and lived alone, with the help of his daughters. His eating habits had been problematic in the past and led to a worrisome weight loss. *Stan*’s daughters had decided to hire a CES^b^ employee. Before receiving the telemonitoring report, the HSCP in charge of *Stan*’s care had planned to ask the CES employee to implement a complete meal preparation occupational routine (ie, when to eat, what to eat, in what quantity, etc). The telemonitoring report showed that *Stan* already ate regularly, 3 times a day. | - “I think we’ve actually decreased a bit. We used to be present, and then we realized that everything was fine. Then we kind of, in terms of giving medication, I think there were things that were given that now are monitored like once a day or two, there. Then there are calls rather than visits. Then, during our checkups, everything is good...Even diet. I think there’s just...in the morning visit I think they check a bit that...in terms of taking meals. But the family continues to prepare meals. Madame organizes her lunch and with the data from the sensors, we were able to see that she is active at regular times for food. And that she uses the microwave more at lunchtime. That she cooks more in the evening. And that the sensor isn’t on the oven’s electricity. It’s only on the door. But, hey, there’s some activity going on.” [HSCP in charge of *Thérèse*, CISSS 3] - “In fact, it confirmed to us that every day, he eats three times a day. So, we know we don’t need to intervene, even when his daughters aren’t there, we know we don’t need to intervene on the habit of eating. We do not need to intervene on the whole ‘what to eat, in quantity and in quality.’” [HSCP in charge of *Stan*, CISSS 3] |
| Substep 4b1. Maintenance of the intervention plan | - Most HSCPs deemed it unnecessary to add an intervention when the telemonitoring report showed that the care recipient performed an ADL inadequately if this ADL was not at the core of their home care challenges (eg, they did not perform desirable activities as often as before). - Occurrences of ADLs recorded: meal preparation=2, sleep=3, hygiene=1, outings=3, and activity level=1. | - In the past, *Christine* did not go out much, and the HSCP in charge of her care at the time was preoccupied. The new HSCP now in charge of *Christine*’s care remained vigilant of their predecessor’s preoccupation. When the telemonitoring report showed a decrease in her outings, the new HSCP discussed the data with *Christine*. | - “Then, at some point, the outings—but I knew she was going out a little less—so I asked her about it...Then, I don’t know if...maybe you weren’t informed, but, basically, she was going out less because there’s work going on right now in the block where she lives. In fact, they used to be able to go inside to the IGA [grocery store], but now they have to go outside. Ah, that’s it. I know that for Madame, she mentioned to me that she found it more difficult.” [HSCP in charge of *Christine*, CIUSSS 1] |
| Substep 4b2. Intervention plan maxed out | - Some HSCPs could not modify an intervention plan that had already reached maximum capacity although the telemonitoring report showed that the care recipient did not perform ADLs adequately to meet their needs (eg, they did not perform desirable activities, did so too rarely, or did so for an insufficient duration). - Occurrences of ADLs recorded: meal preparation=1, sleep=0, hygiene=2, outings=0, and activity level=0. | - Long before his referral to the research project, *Steve*’s home care was very precarious. He had major cognitive difficulties, and he lived alone. He received very few visits from his HSCP because he distrusted most people and did not tolerate their intrusions in his private life. *Steve*’s hygiene was particularly problematic, and his appearance showed signs of neglect. *Steve* rejected all previous attempts by the HSCP to implement services to better support him at home. The telemonitoring report showed minimal movement in the bathroom and the shower. | - “I’d say it (the data) didn’t influence our intervention plan. Because, I mean, it would influence our intervention plan, I don’t want to be negative, but it would influence our intervention plan for his hygiene. I’d suggest he gets assistance again. But knowing that we’re facing a refusal, I mean, I won’t do it. Even if it’s...we know there’s a little movement. A little bit of...he’s getting into the shower, all that, but that it would require a better, how can I put it, assistance from an auxiliary to give him a full shower, a full bath. But given the fact that we’re...as I say, for this gentleman, it’s categorical, it’s a refusal. So, I’m not proposing a new intervention plan...It’ll be a relocation if we judge that, in the end, it’s no longer relevant, it’s become too dangerous.” [HSCP in charge of *Steve*, CISSS 3] |
| Substep 4b3. Addition to the intervention plan | - Most HSCPs added an intervention to their intervention plan when the telemonitoring report showed that the care recipient did not perform ADLs adequately to meet their needs (eg, they did not perform desirable activities or did so for an insufficient duration). - Occurrences of ADLs recorded: meal preparation=1, sleep=0, hygiene=1, outings=0, and activity level=0. | - *Patty*, who had neurocognitive deficits, lived alone, and her eating habits were problematic. While no services were in place yet, the HSCP wanted her to eat 3 meals a day. The telemonitoring report showed that *Patty* ate a simple meal in the morning and cooked once, in the late afternoon. | - “So, it says that she eats a simple meal between 11 AM and noon because she uses everything she needs, and then ‘seems to cook between 4:30 and 6 PM,’ so I have the impression that she eats just twice a day. So, yes, I’d have to see to it that she eats at least three times a day...But like, for her, I’m going to intervene, I’m going to see her, as I was saying, to take a look at her eating routine. So, I’m going to redecide with her, whereas if I hadn’t had that information, I wouldn’t have done it. And since we’re in the prevention business, it’s good to be able to help her.” [HSCP in charge of *Patty*, CISSS 3) |
| Substep 4b4. .Intervention plan put on hold | - Some HSCPs put their intervention plan on hold when the telemonitoring report showed that the care recipient did not perform ADLs adequately to meet their needs (eg, they scarcely performed desirable activities to meet their needs) when the data were not compatible with the services their client received. - Occurrences of ADLS recorded: meal preparation=2, sleep=0, hygiene=3, outings=0, and activity level=0. - Some HSCPs put their intervention plan on hold although the telemonitoring report showed that the care recipient performed ADLs adequately to meet their needs (eg, they regularly performed desirable activities) when the data were not compatible with their observations of their client.   Occurrences of ADLs recorded: meal preparation=1, sleep=0, hygiene=1, outings=0, and activity level=0. | - *Lois*’s eating habits were considered problematic. The HSCP in charge of her care had observed unused food in the refrigerator and had frequently thrown away unopened, expired food. To support her home care, *Lois* received 3 meals per week from a Meals on Wheels program. The HSCP was perplexed when the telemonitoring report showed that she seldom used the microwave to reheat them. - *Louise*, who had neurocognitive disorders, lived alone at home. The habits she reported to her HSCP were unreliable because she did not recognize her difficulties. The HSCP had observed her neglected appearance and, as such, was surprised when the telemonitoring report showed that she showered regularly. | - “In fact, we’d already received two reports...in all, we’d received two reports for the data. And in fact, the first report confirmed most of our information, with the exception of the food, but at that time, she was also often removing the microwave sensors. So, it was during the second report that [the research professional] made a modification so that the sensor would really remain in place at all times. And that’s when we...at that time, we saw that she was using the microwave only three times, I think, on average per month.” [HSCP in charge of *Lois*, CIUSSS 1). - *“*Let’s face it, in [the] first report, there was a lot of activity in the bathroom. Which really surprised us. Because it was as if she got in and out of the shower regularly during the week. But for us, according to our observations, it didn’t quite fit. To finally discuss with [the research professional], see how the sensors were installed, and what was really recorded as activity...So, [the research professional] made another visit to move some sensors around, so that we could get better data...So, after that, he was able to produce a more accurate report.” [HSCP in charge of *Louise*, CISSS 3] |
| 4c. Improve the timing of interventions | - Some HSCPs used the telemonitoring reports to plan their visits and follow-ups with their client when the reports showed that they performed activities regularly (eg, they were active at regular times or they were absent at regular times). | - *Lisa* had Alzheimer disease, lived alone, and already received many home care services. The telemonitoring reports showed that she used her oven at mostly regular hours and went out regularly between 9 and 10 AM. | - “Yes, actually, like you know, we see that the oven, she always uses it around noon. So, for me, I make sure that I won’t arrive at noon exactly, I’ll arrive a little before to be sure that Madame is present to make her meal. Although for the exits, we did not see exactly when she left, but [the research professional] wrote to us: ‘Generally, between 9 AM and 10 AM, she opens the garage door.’ The fact that we know that between 9 AM and then 10 AM, she goes for a walk, so we don’t go there between those hours.” [HSCP in charge of *Lisa*, CISSS 3] |
| 4d. Confirm the delivery of the intervention plan | - Some HSCPs used the telemonitoring reports to check for activity in their client’s home during planned service hours (eg, increased activity on the days services were provided to the care recipient). | - *Peter* had Alzheimer disease, and memory issues made it difficult for him to reliably report whether he had eaten. In the past, weight loss had led the HSCP in charge of his care to implement a stimulation service 3 times a week for 3 h in the afternoon. Upon receiving the first telemonitoring report, the data showed increased activity in the kitchen, 3 times a week, in the afternoon. | • “Then also, we saw, because I can’t remember how long it’s been, but he has an employee who will spend time with him, three hours a day, to encourage him to eat, because at one point he had a weight loss, since he can’t remember if he ate or not. Fact is that we put an employee in place. Then, your detectors picked up that yes, indeed, from such time to such time, on Tuesdays, Wednesdays, Thursdays, there is more movement in the house, so that also allows us to confirm that the employee is indeed there, as he says he is.” [HSCP in charge of *Peter*, CIUSSS 1] |
